# ERα Signaling in a Subset of CXCL12‐Abundant Reticular Cells Regulates Trabecular Bone in Mice

**DOI:** 10.1002/jbm4.10657

**Published:** 2022-06-17

**Authors:** Julia M Scheffler, Karin L Gustafsson, Aidan Barrett, Carmen Corciulo, Christina Drevinge, Alicia M Del Carpio Pons, Piotr Humeniuk, Cecilia Engdahl, Jan‐Åke Gustafsson, Claes Ohlsson, Hans Carlsten, Marie K. Lagerquist, Ulrika Islander

**Affiliations:** ^1^ Centre for Bone and Arthritis Research, Department of Rheumatology and Inflammation Research, Institute of Medicine, Sahlgrenska Academy University of Gothenburg Gothenburg Sweden; ^2^ Centre for Bone and Arthritis Research, Department of Internal Medicine and Clinical Nutrition, Institute of Medicine, Sahlgrenska Academy University of Gothenburg Gothenburg Sweden; ^3^ Center for Nuclear Receptors and Cell Signaling, Department of Biology and Biochemistry University of Houston Houston TX USA; ^4^ Department of Biosciences and Nutrition Karolinska Institute Huddinge Sweden; ^5^ Department of Drug Treatment Sahlgrenska University Hospital, Region Västra Götaland Gothenburg Sweden

**Keywords:** ESTROGEN, GENETIC ANIMAL MODEL, OSTEOIMMUNOLOGY, OSTEOPOROSIS, STROMAL CELL

## Abstract

Estrogen has pronounced effects on the immune system, which also influences bone homeostasis. In recent years, stromal cells in lymphoid organs have gained increasing attention as they not only support the regulation of immune responses but also affect bone remodeling. A conditional knockout mouse model where estrogen receptor alpha (ERα) is deleted in CCL19‐expressing stromal cells (Ccl19‐Cre *ERα* ^*fl/fl*^ mice) was generated and bone densitometry was performed to analyze the importance of stromal cell–specific ERα signaling on the skeleton. Results showed that female Ccl19‐Cre *ERα* ^*fl/fl*^ mice display reduced total bone mineral density and detailed X‐ray analyses revealed that ERα expression in CCL19‐expressing stromal cells is important for trabecular but not cortical bone homeostasis. Further analysis showed that the trabecular bone loss is caused by increased osteoclastogenesis. Additionally, the bone formation rate was reduced; however, the expression of osteoprogenitor genes was not altered. Analysis of the bone marrow stromal cell compartment revealed a deletion of ERα in a subgroup of CXCL12‐abundant reticular (CAR) cells resulting in increased secretion of the pro‐osteoclastogenic chemokine CXCL12. In conclusion, this study reveals the importance of ERα signaling in CAR cells for bone health. © 2022 The Authors. *JBMR Plus* published by Wiley Periodicals LLC on behalf of American Society for Bone and Mineral Research.

## Graphical abstract

Deletion of ERα in CCL19‐expressing stromal cells affects a subpopulation of bone marrow CXCL12‐abundant reticular (CAR) cells.

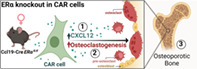

① increased secretion of CXCL12;

② increased osteoclastogenesis;

③ loss of trabecular bone.

Created with BioRender.com.

## 1 Introduction

Osteoporosis is a chronic skeletal disorder common in postmenopausal women. The disease is caused by imbalance in bone remodeling that results in low bone mineral density (BMD) and can ultimately lead to bone fragility.^(^ ^1^ ^)^ Estrogen has a major role in bone homeostasis, and it is well known that estrogen‐containing hormone replacement therapy (HRT) in postmenopausal women attenuates osteoporosis and prevents osteoporotic fractures.^(^ ^2^, ^3^, ^4^ ^)^ However, besides the benefits of HRT, unwanted side effects are reported and a better understanding of the role of estrogen in bone homeostasis is needed to develop improved treatment strategies.^(^ ^5^, ^6^ ^)^

Interactions between immune cells and tissue resident cells in primary and secondary lymphoid organs are essential for a well‐functioning immune system and tissue maintenance. Stromal cells, long ignored as mere scaffolding cells, have gained increasing interest for their role as immunomodulators. It is now well established that besides secreting chemoattractants and cytokines, stromal cells in lymphoid organs provide an important platform for immune cell interaction and activation. Also, the stromal‐/immune‐cell cross‐talk is important for the regulation of immune responses during many physiological and pathological processes, including acute infections as well as chronic diseases like osteoporosis.^(^ ^7^, ^8^ ^)^

In recent years, the field of osteoimmunology has emerged, focusing on the impact of the immune system on bone homeostasis and vice versa. The bone marrow (BM) is home to mesenchymal as well as hematopoietic cell differentiation. Osteoporosis is the result of an imbalance between bone formation by mesenchymal osteoblasts (OBL) and bone resorption by osteoclasts (Oc) of hematopoietic origin.

Mesenchymal BM stromal cells have long been associated with bone homeostasis, and recently a specific population, the CXCL12‐abundant reticular (CAR) cells, has gained increasing focus.^(^ ^9^, ^10^, ^11^ ^)^ Single‐cell RNA sequencing and imaging studies have dissected the CAR cell population into subgroups defined by their expression of signature genes and BM localization. The subgroup osteo‐CAR cells are located in the periarteriolar, nonvascular, and trabecular region, and express osteogenic markers as alkaline phosphatase (*Alpl*) and Osterix (*Sp7*), whereas adipo‐CAR cells express adipogenic markers and are found perisinusoidally. These cells have the potential to differentiate into OBL and adipocytes, respectively.^(^ ^12^, ^13^, ^14^, ^15^, ^16^ ^)^ Furthermore, CXCL12‐positive human BM stromal cells have been demonstrated to give rise to OBL and adipocytes in vitro.^(^ ^17^ ^)^

Estrogen is a known influencer of bone homeostasis. Animal models using ovariectomy (OVX) to mimic postmenopausal osteoporosis are widely used to study the mechanisms involved.^(^ ^18^ ^)^ Conditional knockout mouse models deleting estrogen receptor alpha (ERα) in various mesenchymal cell types further underline the importance of estrogen for bone, since both Paired related homeobox (Prx)1‐Cre and Osterix (Osx)1‐Cre *ER* α knockout mice suffer from bone loss.^(^ ^19^ ^)^ Deletion of ERα in mesenchymal progenitors using Prx1‐Cre mice led to increased osteoclastogenesis, which resulted in cortical bone loss. This was at least partially driven by increased secretion of CXCL12 from CAR cells due to deficient estrogen signaling.^(^ ^20^ ^)^ Furthermore, deletion of CXCL12 in Prx1‐targeted cells resulted in increased total bone turnover and protected against OVX‐induced cortical bone loss.^(^ ^21^ ^)^ In contrast, it was shown that deletion of CXCL12 in Prx1‐ or Osx1‐expressing mesenchymal stem/progenitor cells, but not in osteoblasts, resulted in reduced trabecular bone content in male mice.^(^ ^22^ ^)^ These findings demonstrate the need for more research on how estrogen regulates bone homeostasis through CAR cells.

CCL19 is a chemoattractant known to be expressed by stromal cells to direct the migration of naïve lymphocytes to secondary lymphoid organs and CCL19 was recently also identified to be expressed on CAR cells in the BM stroma.^(^ ^13^, ^14^, ^16^, ^23^ ^)^ To our knowledge, the importance of CCL19‐expressing CAR cells in estrogen‐mediated protection of the skeleton has never previously been investigated. In this article, we studied bone homeostasis in a mouse model where ERα was deleted in CCL19‐expressing stromal cells (Ccl19‐Cre *ERα* ^*fl/fl*^). We show that 3‐month‐old, female Ccl19‐Cre *ERα* ^*fl/fl*^ mice suffer from trabecular but not cortical bone loss. This is mainly induced by increased osteoclastogenesis, resulting in increased bone resorption and accompanied by reduced bone formation. Furthermore, loss of estrogen signaling in CCL19‐expressing stromal cells decreases the CAR cell population but increases the secretion of its signature cytokine CXCL12.

## 2 Materials and Methods

### 2.1 Animals

The experimental procedures were approved by the regional ethical review board in Gothenburg. The mice were housed in a standard animal facility under controlled temperature (22 °C) and photoperiod (12 hours of light and 12 hours of darkness). They were fed a pellet diet (Teklad diet 2016, Envigo, Indianapolis, IN, USA) and tap water *ad libitum*. The *ERα* ^*fl/fl*^ mice were provided by Prof Jan‐Åke Gustafsson (University of Houston, TX, USA) (Supplemental Table S1) and their generation has been described previously.^(^ ^24^ ^)^ The Ccl19‐Cre mice were provided by Prof Burkhard Ludewig (Kantonsspital St. Gallen, Switzerland) (Supplemental Table S1).^(^ ^25^ ^)^ The ROSA26‐EYFP reporter mice were purchased from Jackson Laboratory (Bar Harbor, ME, USA) (Supplemental Table S1). The *ERα* ^*fl/fl*^ mice were crossed with the Ccl19‐Cre mice to obtain Ccl19‐Cre *ERα* ^*fl/fl*^ mice and their littermate controls, *ERα* ^*fl/fl*^ mice. PCR of DNA isolated from lymph nodes (LN) confirmed the deletion of ERα in the Ccl19‐Cre *ERα* ^*fl/fl*^ mice (Supplemental Fig. S1*A*). Uteri weights were determined to ensure that the ERα deletion in CCL19‐expressing cells did not influence systemic estrogen levels. No differences between the genotypes were observed (Supplemental Fig. S1*B*).

For some experiments, Ccl19‐Cre *ERα* ^*+/+*^ mice crossed with the ROSA26‐EYFP reporter mice were used.

For all experiments, 3‐month‐old female mice were euthanized by anesthesia with Ketanest/Dexdomitor (Orion Pharma, Espoo, Finland) injection followed by cervical dislocation. Blood, organs, and bones were collected and used for further analysis.

### 2.2 Dual‐energy X‐ray absorptiometry (DXA)

Analysis of total body areal bone mineral density (aBMD) was performed using Faxitron UltraFocus DXA (Faxitron Bioptics, Tuscon, AZ, USA). The region of interest in the spine was defined as vertebra L_3_ to L_6_.

### 2.3 Peripheral quantitative computed tomography (pQCT)

Computed tomographic scans were performed with the pQCT XCT RESEARCH M (version 4.5B, Norland, Fort Atkinson, WI, USA) operating at a resolution of 70 μm, as described previously.^(^ ^26^ ^)^ Trabecular bone in the distal femur was analyzed ex vivo in a metaphyseal scan and defined as the inner 45% of the total cross‐sectional area.

### 2.4 High‐resolution microCT (μCT)

High‐resolution μCT analyses were performed using Skyscan 1172 scanner (Bruker MicroCT, Aartselaar, Belgium) as previously described.^(^ ^27^ ^)^ Briefly, femur was imaged with an X‐ray tube voltage of 50 kV, a current of 200 μA, and a 0.5 mm aluminum filter. The scanning angular rotation was 180°, and the angular increment was 0.70°. The voxel size was 4.49 μm isotropically. NRecon (version 1.6.9) was used to perform the reconstruction after the scans. In femur, the trabecular bone proximal to the distal growth plate was selected for analyses within a conforming volume of interest (cortical bone excluded) commencing at a distance of 650 μm from the growth plate and extending a further longitudinal distance of 134 μm in the proximal direction. Cortical measurements were performed in the diaphyseal region of the femur starting at a distance of 5236 μm from the growth plate and extending a further longitudinal distance of 134 μm in the proximal direction. In vertebra, the voxel size was 6.41 μm and the trabecular bone in the vertebral body caudal of the pedicles was selected for analysis within a conforming volume of interest (cortical bone excluded) commencing at a distance of 5 μm caudally of the lower end of the pedicles and extending a further longitudinal distance of 224 μm in the caudal direction. Nomenclature, symbols, and units were used as described before.^(^ ^28^ ^)^

### 2.5 ELISA

Peripheral blood samples were collected in clotting activator containing tubes (Microvette 500 Z‐Gel, Sarstedt, Nümbrecht, Germany) and serum was extracted. For BM plasma, femurs and tibias were dissected, ends were cut off, and bones were centrifuged in Eppendorf tubes for 30 seconds at 600*g*, 4°. The BM pellets were resuspended in 150 μL PBS per bone and collected in one tube for each mouse. After centrifugation for 2 minutes, at 600*g*, 4°, the supernatant was collected as BM plasma. The bone resorption marker C‐terminal type I collagen fragments was measured in serum using an ELISA RatLaps kit (CTX‐I, Immunodiagnostic Systems, East Boldon, UK) and procollagen type I N propeptide (PINP, Immunodiagnostic Systems) was analyzed as a serum marker of bone formation. The stromal cell factor C‐X‐C motif chemokine 12 (CXCL12) (R&D Systems, Minneapolis, MN, USA) was measured in serum and bone marrow plasma. All ELISA assays were performed according to the manufacturer's instructions.

### 2.6 qPCR

RNA from cortical bone (tibia), trabecular bone (vertebra), BM, adipocytes, OBL, and BM stromal cells was extracted using TriZol Reagent (Sigma, St. Louis, MO, USA) followed by RNeasy Mini QIAcube Kit (Qiagen, Hilden, Germany). RNA were reversed transcribed into cDNA using the Applied Biosystems High‐Capacity cDNA Reverse Transcription Kit (Applied Biosystems, Waltham, MA, USA). qPCR was run using the StepOnePlus Real‐Time PCR systems (Applied Biosystems). Predesigned probes for *Tnfsf11* (Rankl) (Mm00441908_m1), *Tnfrsf11b* (OPG) (Mm00435452_m1), *Esr1* (ERα) (Mm00433147_m1), *Bglap* (osteocalcin) (Mm03413826_mH), and *Runx2* (Mm00501580_m1) from Applied Biosystems were used. The mRNA abundance of each gene was calculated using the ΔΔCt method and adjusted for expression of 18 S ribosomal RNA (4310893E, Applied Biosystems).

### 2.7 Flow cytometry and magnetic‐activated cell sorting (MACS)

Femurs were flushed with PBS, and erythrocyte lysis with 0.83% ammonium chloride in 0.01 M Tris buffer (Sigma) was performed. For flow cytometry of stromal cells in LN and BM, the organs were digested to obtain single cells.

Inguinal LNs were dissected, torn apart with forceps, and digested for 20 minutes at 37° and 500 rpm shaking in digestion buffer (RPMI, Gibco, Amarillo, TX, USA) with 2% FBS (Sigma), 0.8 mg/mL dispase II (Life Technologies, Carlsbad, CA, USA), 0.2 mg/mL collagenase P (Roche/Sigma, St. Louis, MO, USA) and 0.1 mg/mL DNase (Worthington Biochemical Corp, Lakewood, NJ, USA). After 20 minutes, LN fragments were pipetted up and down with a 1000 μL pipette several times to support digestion. After letting the undigested tissue fragments settle for 30 seconds, the supernatant was collected in a fresh tube and digestion was stopped by washing with RPMI medium containing 2% FBS. Undigested LN fragments were digested one more time with digestion buffer at 37° for 10 minutes. After 10 minutes, LN fragments were vigorously pipetted up and down several times to support digestion. The harvest of the supernatant was repeated as before. If needed, the remaining tissue fragments were digested one more time for 10 minutes. Now, the LN suspension was vigorously pipetted up and down every 2 minutes during digestion to obtain single cells. The harvest of the supernatant was repeated as before and the washed cells were joined. Negative selection by MACS was performed to discard CD45‐positive cells and erythrocytes (Ter119^+^) from the LN cells obtained after digestion. The cells were stained with the respective antibody‐coupled magnetic beads (Supplemental Table S2), loaded on a LS column (Miltenyi, Bergisch Gladbach, Germany), and the flow through CD45/Ter119‐negative population was collected for flow cytometry analysis.

To obtain BM stromal cells, both femurs and tibias from the mouse were dissected, the ends were cut off, and BM was flushed out using RPMI with 2% FBS. The remaining bones were cut into pieces. BM and bone pieces were joined and digested in digestion buffer (RPMI with 2% FBS, 0.8 mg/mL dispase II, 1 mg/mL collagenase 2 [Worthington Biochemical Corp] and 0.1 mg/mL DNase) for 45 minutes at 37° and 500 rpm shaking. The digestion was stopped by washing with PBS. All obtained cell suspensions were filtered through a 70 μm cell strainer before staining for flow cytometry analysis.

The cells were first stained with eBioscience Fixable Viability Dye eFluor 780 (Thermo Fisher Scientific, Waltham, MA, USA), followed by incubation with Fc‐gamma receptor block (Becton Dickinson [BD], Franklin Lakes, NJ, USA). Afterward, the cells were stained with fluorescence‐labeled antibodies (Supplemental Table S2). Fluorescence minus one (FMO) and unstained samples were used as controls. The cells were acquired using FACSVerse (BD). The data were analyzed with FlowJo software Version 10 (FlowJo, Ashland, OR, USA).

### 2.8 Calcein labeling

Twelve‐week‐old female mice of both genotypes were injected twice with calcein (green) (Supplemental Table S3; Sigma): 8 days and 1 day before death to stain for bone formation. Femurs were prepared for sectioning as described previously,^(^ ^29^ ^)^ and 5 μm longitudinal sections were cut. The paraffin sections were hydrated using xylene (Sigma) and alcohol series and then stained for 3 minutes with 0.1% calcein blue (Sigma) solution to stain bone. Afterward, dehydration steps through alcohol and xylene were performed and sections were mounted. Confocal tilescan images of the distal trabecular and endocortical area were taken with a 20× objective using Leica SP8 confocal microscope equipped with a 488 nm blue laser and a 405 nm UV laser, HyD detectors (Leica, Wetzlar, Germany) and the Leica Application Suite X software (Leica). Blinded images were analyzed using the bone histomorphometry software CalceinHisto.^(^ ^30^ ^)^ Mineral apposition rate (MAR) was calculated as the distance between the two calcein incorporations divided by 7 days.

### 2.9 Osteoblast and BM stromal cell culture

Femurs and tibias were dissected from female mice and BM cells were flushed out with basic media (αMEM, Gibco) supplemented with 1% 100 U/penicillin, 100 μg/mL streptomycin (Gibco), 1% Glutamax (Gibco), and 10% FBS (Sigma). Single cells were filtered through a 70 μm cell strainer and seeded (one well/mouse on a 6‐well plate). After 2 days, cells were collected in media by scraping and counted. Cells were drop‐seeded at a concentration of 10,000 cells/μL (76 μL/well on a 12‐well plate) and covered with 1 mL media after 5 minutes. After reaching 70% confluency, differentiation factors were added to the media for osteoblast differentiation: β‐glycerophosphate and ascorbic acid (Supplemental Table S3). For BM stromal cells, basic media without differentiation factors was used. The cell culture medium was changed every third day. Osteoblasts started to differentiate after 5 days of culture, and all cells were harvested at 12 days of culture.

### 2.10 Adipocyte culture

Single cells from BM were obtained as described above. Cells were seeded at a density of 1 × 10^6^ cells/cm^2^ in basic media. After 3 days, medium was changed and cells were cultured until 80% confluency. Differentiation was induced by adding insulin, dexamethasone, and indomethacin (Supplemental Table S3) to the basic media. Afterward, the differentiation medium was changed every 3 days and cells were harvested on day 12 after differentiation induction.

### 2.11 Tartrate‐resistant acid phosphatase (TRAP) staining

Femurs were fixed in 4% formaldehyde for 2 days, decalcified with 10% EDTA for 14 days, and embedded in paraffin. Sections (8 μm) were rehydrated using xylene (Sigma) and alcohol series. Hydrated slides were incubated in 0.2 M acetate buffer, pH = 5.0 (Sigma) for 1 to 1.5 hours at room temperature. Then the slides were transferred into prewarmed TRAP buffer, pH 5.0 (0.05 M acetate buffer, 0.03 M sodium tartrate, 0.1 mg/mL Naphtol AS‐MX phosphate, 0.1% Triton X‐100, 0.3 mg/mL Fast Red Violet LB stain (Sigma) and incubated for 30 minutes at 37°. Slides were washed with water and counterstained with 0.05% Fast Green (Sigma) for 45 seconds. After washing with water, the slides were dehydrated and mounted. Pictures of the distal trabecular area of the femur were taken with a 20× objective of an EVOS XL Core Imaging System (Thermo Fisher Scientific). Stitching was performed with Adobe Photoshop 21.1.0 (Adobe, San Jose, CA, USA), and quantification of TRAP staining was performed with bone histomorphometry software TrapHisto.^(^ ^30^ ^)^

### 2.12 Osteoclast culture from bone marrow cells

Bone marrow cells (BMC) were flushed from both femurs and tibias with αMEM media (Gibco) supplemented with 1% 100 U/penicillin, 100 μg/mL streptomycin (Gibco), 1% Glutamax (Gibco), 50 ng/mL gentamycin (Gibco), and 10% FBS (Sigma) using a syringe and needle. Cells were washed once with basic media and counted. A total of 1 × 10^6^ BMC/cm^2^ were plated in basic media supplemented with 30 ng/mL MCSF and 4 ng/mL RANKL (both from (R&D Systems) on a 96‐well plate. Medium was changed after 3 and 5 days. On day 7, cells were fixed and stained with the Acid Phosphatase, Leukocyte (TRAP) Kit (Sigma) according to the manufacturer's instructions. Stained osteoclasts were counted manually, and representative pictures were taken using a Leica DMR microscope equipped with a Leica DFC420 C camera using the Leica Application Suite V3 software (Leica).

### 2.13 Immunofluorescence

Femurs were fixed in 4% PFA overnight and decalcified in 10% EDTA for 4 days. After dehydration with 10% and 30% sucrose, bones were embedded in OCT and sectioned (20 μm). Sections were blocked with blocking buffer (0.2% Triton‐X 100, and 10% goat serum in PBS) for 1 hour at room temperature. Primary antibody incubation was performed overnight at 4° in blocking buffer. Ebf3 antibody (Abcam, Cambridge, UK) was used in a 1:100 dilution. After primary antibody incubation, sections were washed for 2 hours in PBS at room temperature and then incubated with an Alexa647‐conjugated goat anti‐rabbit antibody (1:1000, Invitrogen) in PBS. After washing with PBS, sections were mounted with Prolong Diamond Antifade mount (Thermo Fisher Scientific). Images were taken with a SP8 confocal (Leica) using the Leica Application Suite X software (Leica). Images were processed with Fiji.^(^ ^31^ ^)^

### 2.14 Statistical analyses

Statistical analyses were performed using GraphPad Prism (GraphPad, La Jolla, CA, USA; version 9.2). Results are presented as box plots with median, interquartile range, and max and min values. All data points are plotted and normal distribution was analyzed using the Kolmogorov–Smirnov (*n* > 5) or Shapiro–Wilk (*n* < 5) test depending on the group size. Unpaired two‐tailed Student's *t* test was used for comparison of two independent groups when the data was normally distributed. The nonparametric Mann–Whitney *U* test was used when normal distribution was not present. A value of *p* < 0.05 was considered significant and *p* values are plotted.

## 3 Results

### 3.1 ERα signaling in CCL19‐expressing stromal cells is important for trabecular bone homeostasis

**Fig. 1:**
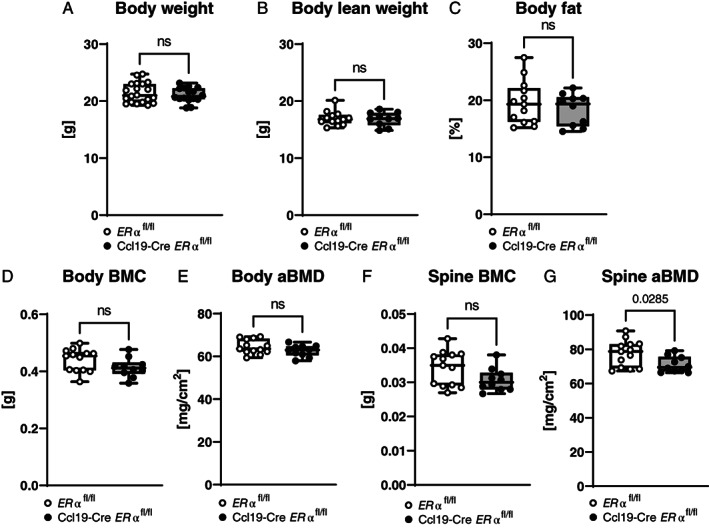
ERα signaling in CCL19‐positive stromal cells affects bone mineral density in the spine. (*A*) Body weight of 12‐week‐old female littermate control *(ERα* ^*fl/fl*^) and Ccl19‐Cre *ERα* ^*fl/fl*^ mice at termination day. *n* = 16–20. (*B–G*) Dual‐energy X‐ray absorptiometry (DXA) measurements of (*B*) whole body lean weight, (*C*) whole body fat, (*D*) whole body bone mineral content (BMC), (*E*) total body areal bone mineral density (aBMD), (*F*) lumbar spine BMC, and (*G*) lumbar spine aBMD. Data from two independent pooled experiments are presented. *n* = 10–13; Student's *t* test: *p* < 0.05, ns = nonsignificant.

**Fig. 2:**
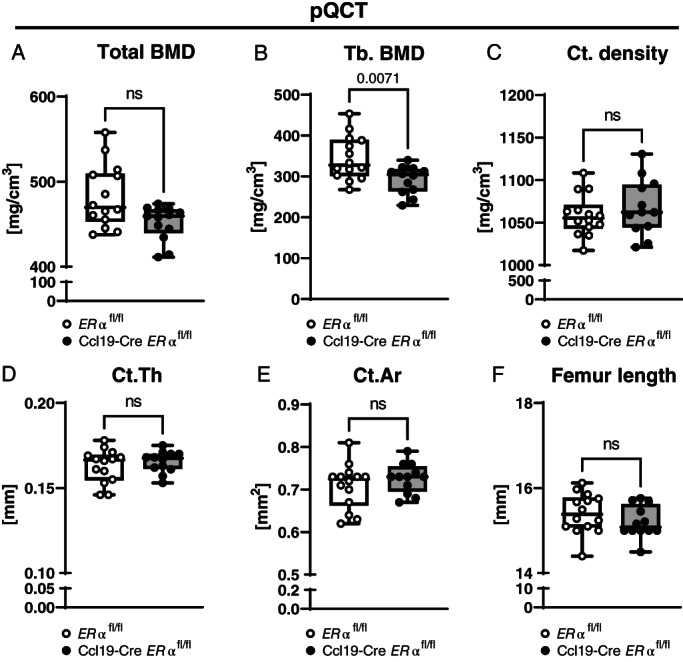
ERα in CCL19‐positive stromal cells regulates trabecular bone mineral density in femur. Femurs of 12‐week‐old female littermate control (*ERα* ^*fl/fl*^) and Ccl19‐Cre *ERα* ^*fl/fl*^ mice were analyzed by peripheral quantitative computed tomography (pQCT). (*A*) Total bone mineral density (BMD), (*B*) trabecular (Tb.) BMD, (*C*) cortical (Ct.) density, (*D*) cortical thickness (Ct.Th), (*E*) cortical area (Ct.Ar), and (*F*) femur length are shown. Data from two independent pooled experiments are presented. *n* = 12–14; (*A*) Mann–Whitney test, (*B–D*) Student's *t* test: *p* < 0.05, ns = nonsignificant.

**Fig. 3:**
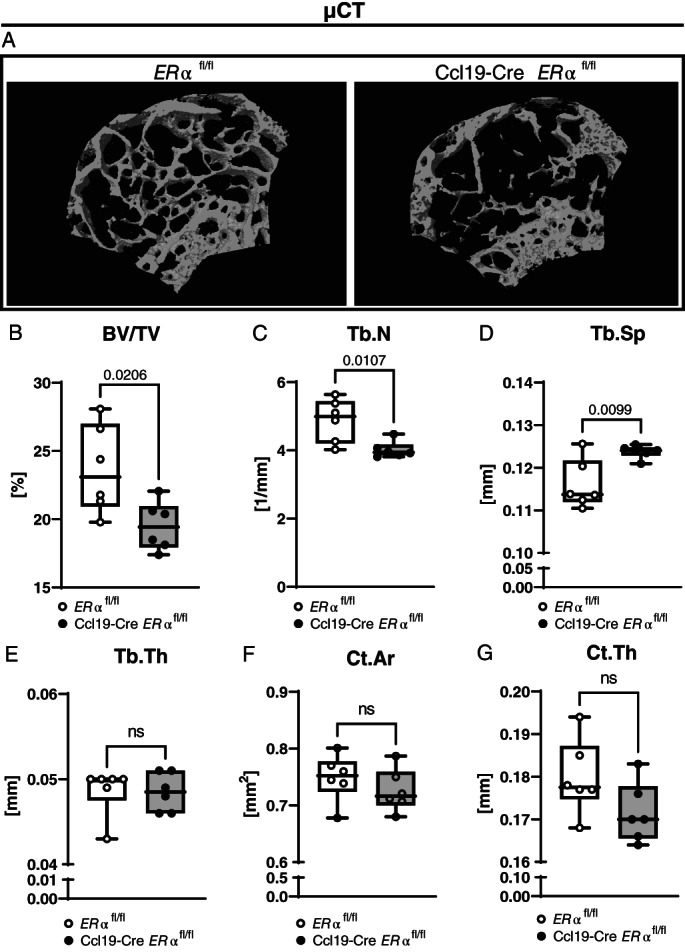
Trabecular bone homeostasis is dependent on ERα signaling in CCL19‐positive stromal cells. Femurs of 12‐week‐old female littermate control (*ERα* ^*fl/fl*^) and Ccl19‐Cre *ERα* ^*fl/fl*^ mice were analyzed by high‐resolution microCT (μCT). (*A*) A representative three‐dimensional reconstruction of the trabecular region proximal to the distal growth plate for each genotype is shown. (*B*) Trabecular bone volume relative to tissue volume (BV/TV), (*C*) trabecular number (Tb.N), (*D*) trabecular separation (Tb.Sp), (*E*) trabecular thickness (Tb.Th), (*F*) cortical area (Ct.Ar), and (*G*) cortical thickness (Ct.Th) was measured. *n* = 6; (*B–D, F–G*) Student's *t* test, (*E*) Mann–Whitney test: *p* < 0.05, ns = nonsignificant.

In a first approach, DXA was performed on 12‐week‐old female mice to characterize body composition of the newly established Ccl19‐Cre *ERα* ^*fl/fl*^ mouse strain. *ERα* ^*fl/fl*^ mice were used as littermate controls. No differences between the genotypes were found for body weight, body lean weight, percentage of body fat, total body BMC, total body aBMD, and spine BMC (Fig. 1*A–F*). However, aBMD of the spine was reduced in Ccl19‐Cre *ERα* ^*fl/fl*^ mice (Fig. 1*G*). To further investigate the bone composition, pQCT of femurs from 12‐week‐old female mice was performed. The analyses showed a significant reduction of trabecular BMD in Ccl19‐Cre *ERα* ^*fl/fl*^ mice compared with controls, whereas cortical parameters as well as bone length were unaffected (Fig. 2*A–F*). Next, μCT of femurs was used to analyze bone microarchitecture in both genotypes. Representative images of the trabecular region at the distal epiphysis of the femur are shown in Fig. 3*A* and measurements of bone volume fraction (BV/TV), trabecular number (Tb.N), and trabecular separation (Tb.Sp) confirm the loss of trabecular bone in Ccl19‐Cre *ERα* ^*fl/fl*^ mice compared with control mice (Fig. 3*B–D*). No differences in trabecular thickness (Tb.Th) or cortical area (Ct.Ar) were observed between the genotypes, but a tendency of decreased cortical thickness (Ct.Th) was observed in the Ccl19‐Cre *ERα* ^*fl/fl*^ mice (Fig. 3*E–G*). In addition to femur, the lumbar vertebra L_5_ was analyzed by μCT and confirmed the finding that Ccl19‐Cre *ERα* ^*fl/fl*^ mice have reduced trabecular bone compared with control mice (Supplemental Fig. S2 *A–D*). Measurements of the bone formation marker procollagen type I N propeptide (PINP) and the bone resorption marker C‐terminal type I collagen fragments (CTX‐I) in serum revealed no differences (Supplemental Fig. S2 *E*, *F*).

No bone phenotype for Ccl19‐Cre mice has been described, but to ensure that the trabecular bone phenotype observed in Ccl19‐Cre *ERα* ^*fl/fl*^ mice is not due to the Ccl19‐Cre strain, femurs of female 12‐week‐old *ERα* ^*+/+*^ and Ccl19‐Cre *ERα* ^*+/+*^ mice were analyzed by pQCT. No difference in trabecular BMD between the genotypes were observed (Supplemental Fig. S2 *G*).

To exclude the possibility that any cell types other than BM stromal cells were targeted in the Ccl19‐Cre *ERα* ^*fl/fl*^ mice, qPCR for *Esr1* (ERα) in various bone‐related tissues was performed. No deletion of ERα in trabecular and cortical bone, BM, or in vitro cultured osteoblasts and adipocytes was detected (Supplemental Fig. S1 *C–G*). However, an approximately 50% reduction of *Esr1* expression was observed in in vitro cultured BM stromal cells (Supplemental Fig. S1 *H*).

In summary, female Ccl19‐Cre *ERα* ^*fl/fl*^ mice display defects in bone homeostasis, which leads to reduced trabecular bone.

### 3.2 Loss of trabecular bone in Ccl19‐Cre *ERα* ^*fl*^ ^*/fl*^ mice is caused by increased osteoclastogenesis and reduced bone formation

**Fig. 4:**
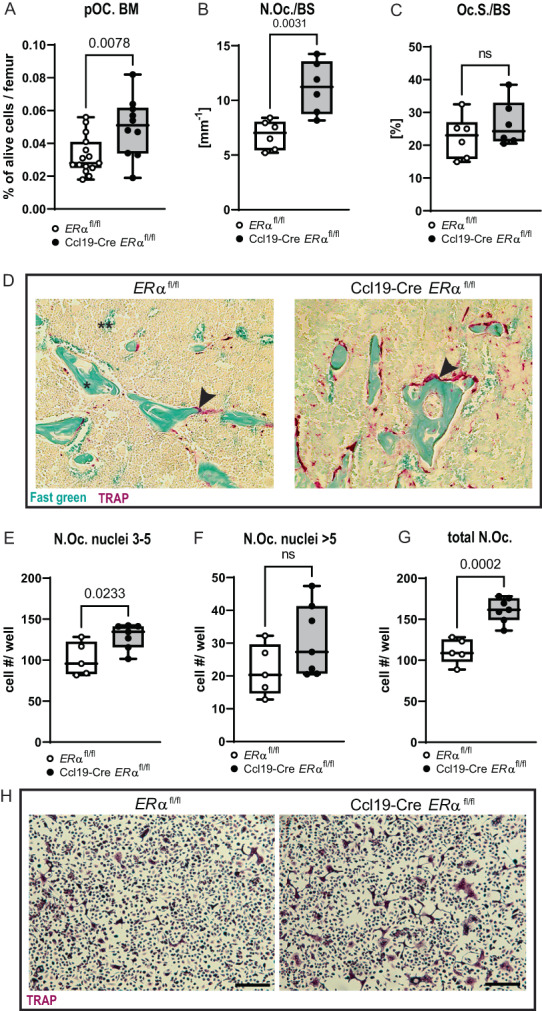
Osteoclastogenesis is regulated by ERα‐signaling in CCL19‐expressing stromal cells. Osteoclastogenesis in 12‐week‐old female littermate control (*ERα* ^*fl/fl*^) and Ccl19‐Cre *ERα* ^*fl/fl*^ mice was analyzed in bone marrow (BM) and the distal trabecular region of femur. (*A*) Flow cytometry was performed on BM cells. Pre‐osteoclasts (pOc.) were defined as MCSFR^+^CD11b^+^RANK^+^ cells and are shown as percentage of alive cells. Data from three independent pooled experiments are presented. *n* = 14–17. Femur sections were stained for tartrate‐resistant acid phosphatase (TRAP) and (*B*) the number of osteoclasts per bone surface (N.Oc./BS), and (*C*) osteoclast surface over bone surface (Oc.S./BS) were quantified at the trabecular region proximal to the distal growth plate. *n* = 6. (*D*) Representative pictures of TRAP staining (red) in the trabecular region for both genotypes are shown. Arrowheads indicate osteoclasts on the bone surface. *Bone is stained green; **background; scale = 100 μm. (*E–G*) In vitro cultures of osteoclasts from bone marrow cells were stained with TRAP, and osteoclast numbers were analyzed according to the number of nuclei as indicated. *n* = 5–7. (*H*) Representative pictures of TRAP‐stained (purple) osteoclast cultures for both genotypes are shown. Scale bar = 200 μm; Student's *t* test: *p* < 0.05, ns = non‐significant.

To understand whether the trabecular bone loss in Ccl19‐Cre *ERα* ^*fl/fl*^ mice is due to increased bone resorption, osteoclastogenesis was investigated. Flow cytometry of BM from Ccl19‐Cre *ERα* ^*fl/fl*^ mice and controls was performed. Although no differences in the BM cell numbers and major myeloid cell populations were detected (Supplemental Fig. S3 *A–E*), the level of pre‐osteoclasts (pOc.) was elevated in Ccl19‐Cre *ERα* ^*fl/fl*^ mice (Fig. 4*A*). In line with this finding, further histological analyses revealed increased numbers of tartrate‐resistant acid phosphatase (TRAP)‐positive osteoclasts on the trabecular bone surface (N.Oc./BS), whereas the osteoclast surface per bone surface (Oc.S./BS) was unaffected (Fig. 4*B–D*), indicating increased osteoclastogenesis in the Ccl19‐Cre *ERα* ^*fl/fl*^ mice. Furthermore, in vitro osteoclast differentiation from BM cells of 12‐week‐old female Ccl19‐Cre *ERα* ^*fl/fl*^ and control mice was performed, and the number of TRAP‐positive osteoclasts was quantified. Whereas the number of osteoclasts with three to five nuclei was significantly increased in cultures from Ccl19‐Cre *ERα* ^*fl/fl*^ mice, the number of osteoclasts with more than five nuclei did not differ between the genotypes (Fig. 4*E, F*). In total, the overall number of in vitro differentiated osteoclasts was increased in Ccl19‐Cre *ERα* ^*fl/fl*^ mice compared with controls (Fig. 4*G*). Representative pictures of quantified TRAP‐stained osteoclast cultures from both genotypes are shown in Fig. 4*H*.

**Fig. 5:**
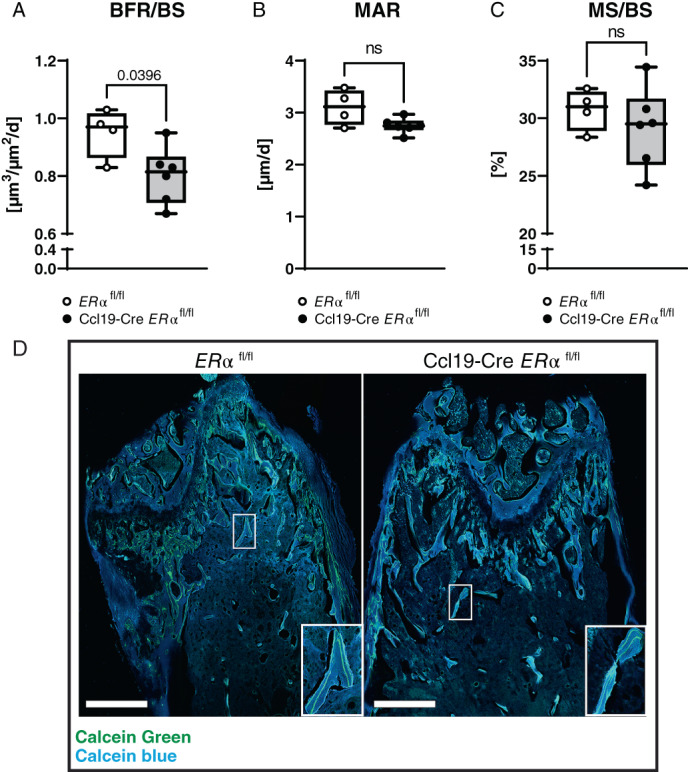
Trabecular bone formation is affected by ERα signaling in CCL19‐positive stromal cells. Bone formation in the distal trabecular region of femurs of 12‐week‐old female littermate control (*ERα* ^*fl/fl*^) and Ccl19‐Cre *ERα* ^*fl/fl*^ mice was analyzed by calcein incorporation. (*A*) Bone formation rate per bone surface (BFR/BS), (*B*) mineral apposition rate (MAR), and (*C*) mineralizing surface per bone surface (MS/BS) were quantified in the trabecular region proximal to the distal growth plate. *n* = 4–6. (*D*) Representative confocal tile scan images of calcein incorporation (green) in the trabecular region for both genotypes are shown. Bone is stained with calcein blue. White squares on the lower right show magnifications. Scale bar = 500 μm; Student's *t* test: *p* < 0.05, ns = nonsignificant.

Additionally, bone formation was investigated. Female mice were injected with two pulses of calcein before euthanization. The distal femur was sectioned and the trabecular region underneath the growth plate was analyzed. The trabecular bone formation rate per bone surface (BFR/BS) was significantly reduced in Ccl19‐Cre *ERα* ^*fl/fl*^ mice, whereas the trabecular mineral apposition rate (MAR) and ratio of mineralizing surface to bone surface (MS/BS) only showed a nonsignificant tendency toward reduction in Ccl19‐Cre *ERα* ^*fl/fl*^ mice (Fig. 5*A–C*). Representative pictures of the quantified trabecular regions for both genotypes are shown in Fig. 5*D*. Furthermore, the endocortical MAR of the distal femur was assessed, but no differences were detected between the genotypes (Supplemental Fig. S4 *A*). These data indicate a reduction in trabecular bone formation of Ccl19‐Cre *ERα* ^*fl/fl*^ mice.

**Fig. 6:**
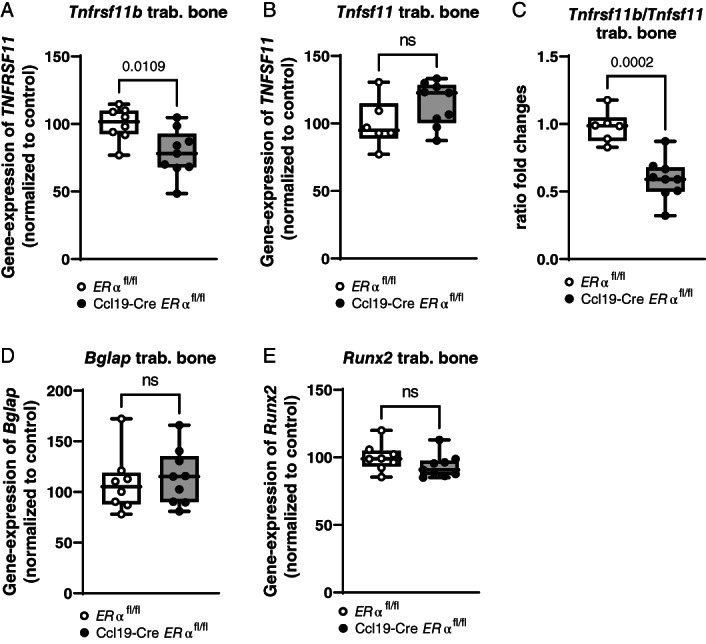
ERα signaling on CCL19‐positive stromal cells regulates expression of bone resorption markers on trabecular bone. Gene expression in trabecular (trab.) bone (vertebra L_3_ and L_6_) of 12‐week‐old female littermate control (*ERα* ^*fl/fl*^) and Ccl19‐Cre *ERα* ^*fl/fl*^ mice was analyzed. Results are shown in percentage normalized to mean expression of *ERα* ^*fl/fl*^ controls: (*A*) *Tnfrsf11b* (OPG), (*B*) *Tnfsf11* (RANKL), (*C*) ratio of *Tnfrsf11b* and *Tnfsf11*, (*D*) *Bglap* (osteocalcin), and (*E*) *Runx2* were calculated. *n* = 6–9; Student's *t* test: *p* < 0.05, ns = nonsignificant.

Further analysis of the expression of bone‐related genes in trabecular bone revealed a significant reduction in the expression of osteoprotegerin (OPG; *Tnfrsf11B*) and a significantly reduced OPG to receptor activator of nuclear factor kappa‐Β ligand (RANKL; *Tnfsf11*) ratio in Ccl19‐Cre *ERα* ^*fl/fl*^ mice (Fig. 6*A–C*) supporting the previous results of increased osteoclastogenesis presented in Fig. 4. In cortical bone, expression of OPG and RANKL were reduced in Ccl19‐Cre *ERα* ^*fl/fl*^ mice, but the ratio was unaltered (Supplemental Fig. S4 *B–D*). The bone formation markers osteocalcin (*Bglap*) and Runt‐related transcription factor 2 (*Runx2*) were unchanged in trabecular (Fig. 6 *D*, *E*) as well as cortical bone (Supplemental Fig. S4 *E*, *F*). Besides bone cells like osteoblasts and osteocytes, B and T cells are major sources of RANKL in the BM.^(^ ^32^ ^)^ However, flow cytometry analysis of the expression of RANKL on T and B cells in BM showed no differences between the genotypes (Supplemental Fig. S5 *A–F*).

In summary, female 12‐week‐old Ccl19‐Cre *ERα* ^*fl/fl*^ mice display increased osteoclastogenesis and reduced bone formation accompanied by reduced OPG to RANKL ratio but no changes in the expression of bone formation markers.

### 3.3 Ccl19‐Cre *ERα* ^*fl*^ ^*/fl*^ mice show a deletion of ERα in a subgroup of CXCL12 abundant reticular (CAR) cells, which leads to increased levels of CXCL12

**Fig. 7:**
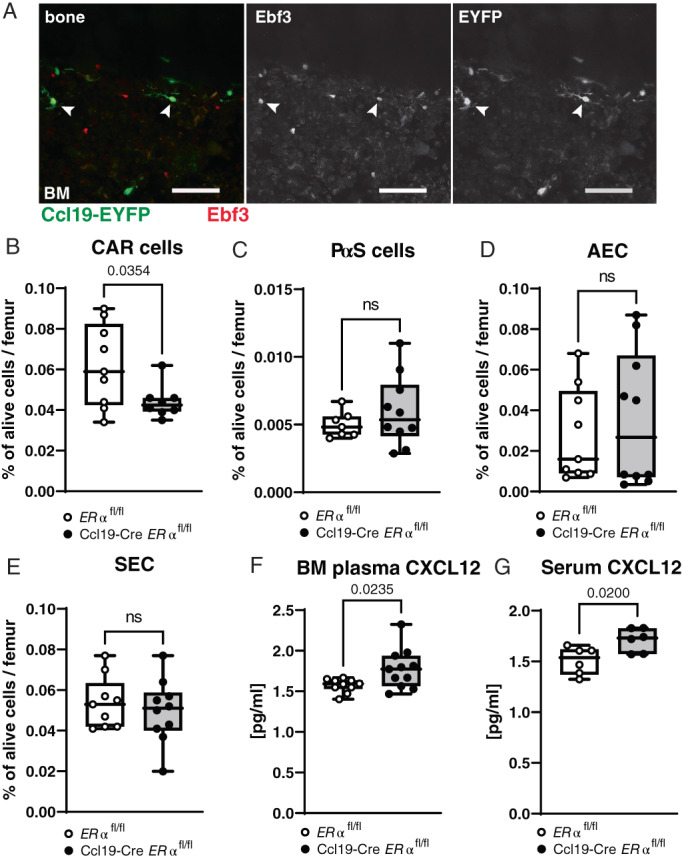
ERα expression in CCL19‐positive CAR cells regulates CAR cell homeostasis and CXCL12 secretion. (*A*) Femur sections of 12‐week‐old female EYFP Ccl19‐Cre *ERα* ^*+/+*^ mice were stained for the transcription factor Ebf3 (red), a CXCL12‐abundant reticular (CAR) cell marker. Endogenous enhanced yellow fluorescent protein (EYFP) signal (green) indicates CCL19‐expressing stromal cells. Left panel shows the merged image in color. Arrows indicate double‐positive stromal cells. Middle and right panels show Ebf3 and EYFP signal, respectively. Scale bar = 50 μm. (*B–E*) Flow cytometry analysis of bone marrow (BM) stromal cells in 12‐week‐old female littermate control (*ERα* ^*fl/fl*^ *)* and Ccl19‐Cre *ERα* ^*fl/fl*^ mice. (*B*) CAR cells, (*C*) Platelet‐derived growth factor receptor (PDGFR)‐α^+^Sca1^+^ cells (PαS), (*D*) arterial endothelial cells (AEC), and (*E*) sinusoidal endothelial cells (SEC) are shown in percentage of alive cells. *n* = 9–10. (*F–G*) Quantification of CXCL12 protein levels in (*F*) BM plasma, *n* = 9–10, and (*G*) blood serum, *n* = 6. (*B, C, E–G*) Student's *t* test, (*D*) Mann–Whitney test: *p* < 0.05, ns = nonsignificant.

As mentioned above, qPCR revealed reduced ERα expression in in vitro cultured BM stromal cells from Ccl19‐Cre *ERα* ^*fl/fl*^ mice (Supplemental Fig. S1 *H*). Literature search confirmed that CCL19 is expressed in the BM by several stromal cell types and especially by CAR cells.^(^ ^13^, ^14^, ^16^ ^)^ To confirm the hypothesis that CAR cells are targeted in the Ccl19‐Cre mice, we used a reporter system in which all CCL19‐expressing cells also express the fluorescent marker EYFP. The Ccl19‐Cre mice were originally designed to target fibroblastic reticular cells (FRC) located in lymph nodes (LN), and as expected, the EYFP Ccl19‐Cre *ERα* ^*+/+*^ mice show an EYFP‐positive population restricted to FRCs in LN (Supplemental Fig. S6 *A*).^(^ ^25^ ^)^ Next, the BM of these mice was analyzed by flow cytometry for EYFP‐positive stromal cells. BM stromal cells were gated as described by Helbling and colleagues^(^ ^16^ ^)^ and approximately 10% of the gated CAR cells were found to be positive for EYFP (Supplemental Fig. S6 *B*). Additionally, confocal imaging confirmed that the EYFP‐positive stromal cells in BM also express the transcription factor Ebf3 (white arrow heads, Fig. 7*A*), which recently was described to be specifically expressed by CAR cells.^(^ ^33^ ^)^ This leads us to the conclusion that Ccl19‐Cre *ERα* ^*fl/fl*^ mice have a deletion of ERα in a subpopulation of the CAR cells.

Next, the BM stromal cell compartment of female Ccl19‐Cre *ERα* ^*fl/fl*^ and control mice was analyzed by flow cytometry. Ccl19‐Cre *ERα* ^*fl/fl*^ mice displayed reduced levels of CAR cells, but no differences in the other types of stromal cells populating the BM (Platelet‐derived growth factor receptor [PDGFR]‐α^+^Sca1^+^ [PαS] cells, arterial endothelial cells [AEC], and sinusoidal endothelial cells [SEC]) compared with controls were found (Fig. 7*B–E*). CXCL12 is the signature cytokine secreted by CAR cells and this chemokine is known to be important, not only in hematopoiesis but also as a marker for the severity of osteoporosis in postmenopausal women.^(^ ^34^, ^35^ ^)^ Indeed, analysis of BM plasma and blood serum revealed increased protein levels of CXCL12 in female Ccl19‐Cre *ERα* ^*fl/fl*^ mice compared with controls (Fig. 7 *F*, *G*).

Taken together, the results indicate that Ccl19‐Cre *ERα* ^*fl/fl*^ mice have a deletion of ERα in a group of CAR cells, which leads to reduced frequency of the CAR cell population in BM but increased secretion of CXCL12.

## 4 Discussion

In this study, we show that ERα signaling in CCL19‐expressing stromal cells is crucial for trabecular bone homeostasis. Furthermore, in line with previously published results showing that CAR cells express CCL19, these data propose that conditional gene deletion with the Ccl19‐Cre mouse model also affects BM stromal cell populations.^(^ ^13^, ^14^, ^16^ ^)^ Concluding, we state that in the Ccl19‐Cre *ERα* ^*fl/fl*^ mice, deletion of ERα in a subgroup of CAR cells results in elevated secretion of CXCL12, increased osteoclastogenesis, and consequently loss of trabecular bone.

The Ccl19‐Cre mouse model is well described and has been widely used to elucidate the role of stromal cells in various organs, during lymphoid organ development as well as disease progression, but no studies concerning BM stromal cells and effects on the skeleton using this model have been performed previously.^(^ ^25^, ^36^, ^37^, ^38^ ^)^ Estrogen and its receptor ERα, on the other hand, are known to be crucial for bone homoeostasis, but still much information regarding the underlying mechanisms for these effects is lacking.^(^ ^39^, ^40^, ^41^ ^)^ Deletion of ERα in CCL19‐positive stromal cells of female mice resulted in an osteoporotic bone phenotype and, more specifically, to increased resorption of trabecular bone. Because osteoblasts and stromal cells as well as adipocytes and chondrocytes share a common progenitor, it was crucial to exclude off‐target deletions of ERα in those tissues.^(^ ^15^ ^)^ No significant reduction of ERα gene expression in bone, cultured osteoblasts, or adipocytes was detected, except for in cultured BM stromal cells. This proposes that the trabecular bone loss observed is not due to deletion of ERα signaling in bone but an effect mediated via the BM stroma.

Recent studies deciphering the complexity of the BM stromal cell compartment revealed that CCL19 is expressed in CAR cells.^(^ ^13^, ^14^, ^16^ ^)^ Knowing that ERα expression in BM stromal cells from Ccl19‐Cre *ERα* ^*fl/fl*^ mice was significantly reduced, we speculated that the Cre expression in Ccl19‐Cre strain might also be present in CCL19‐positive CAR cells, leading to a deletion of ERα signaling in those cells. To prove this, an EYFP reporter strain was crossed into the Ccl19‐Cre *ERα* ^*+/+*^ mice, and it could be shown that approximately 10% of the CAR cells express EYFP. Furthermore, BM sections from the EYFP Ccl19‐Cre *ERα* ^*+/+*^ mice revealed a co‐expression of endogenous EYFP signal and Ebf3, a CAR cell–specific transcription factor, confirming the flow cytometer data.^(^ ^33^ ^)^ This means that the Ccl19‐Cre *ERα* ^*fl/fl*^ mice have a deletion of ERα in a group of CAR cells leading to a decrease of the CAR cell compartment, while arterial and sinusoidal endothelial cells as well as the PαS cells were unaffected. It has been described previously that deletion of ERα can affect cell proliferation and survival, which might explain this observation.^(^ ^42^ ^)^ This is of consequence when considering that CAR cells also are potential osteogenic progenitors.^(^ ^11^ ^)^ The CAR cell subgroup, osteo‐CAR cells, express osteogenic markers like osterix, alkaline phosphatase, and osteopontin and are located around arterioles or in nonvascular regions of the BM.^(^ ^13^, ^14^, ^43^ ^)^ However, whether the deletion of ERα in certain CAR cells affects the differentiation potential of osteo‐CAR cells remains to be elucidated.

Mechanistically, CAR cells are crucial for hematopoiesis by secreting CXCL12, a major hematopoietic stem cell niche factor.^(^ ^44^ ^)^ Besides its function in hematopoiesis, CXCL12 is known to be a potent chemoattractant for pre‐osteoclasts to bone and supports their migration to BM sites for osteoclast differentiation.^(^ ^45^, ^46^, ^47^ ^)^ In several skeletal diseases where a bias toward postmenopausal women is found, eg, rheumatoid arthritis or osteoarthritis, CXCL12 levels are elevated locally in the joints as well as in peripheral blood.^(^ ^48^, ^49^ ^)^ Also, elevated plasma levels of CXCL12 are a marker for disease severity in women with postmenopausal osteoporosis.^(^ ^34^ ^)^ In mice, deletion of ERα in mesenchymal cell progenitors using the Prx1‐Cre mouse leads to elevated expression of CXCL12 by BM stromal cells, indicating an inhibitory effect of estrogen on CXCL12 expression and secretion. In that model, the resulting osteoclastogenesis mainly affects cortical bone in 8‐week‐old female mice. However in 12‐week‐old female ERα^f/f^;Prx1‐Cre mice, a decrease of trabecular number and an increase in trabecular separation is shown in addition to the reduced cortical thickness of femur.^(^ ^19^, ^20^ ^)^ In this study, we show that the levels of CXCL12 in BM and blood was elevated in Ccl19‐Cre *ERα* ^*fl/fl*^ mice and the resulting osteoclastogenesis mostly affects trabecular bone in adult (3‐month‐old) female mice with only a nonsignificant tendency toward reduced cortical area and thickness. Analyses of cortical bone MAR as well as the ratio between OPG and RANKL expression in cortical bone revealed no differences between the genotypes. However, it is possible that analyses conducted on older mice could reveal significant reductions also in the cortical bone compartment of Ccl19‐Cre *ERα* ^*fl/fl*^ mice. Naturally, the discrepancy in effects on cortical bone between this study and other publications could also be related to the different mouse models used.

In summary, we show that deletion of ERα in CCL19‐expressing BM stromal cells partially affects CAR cell homeostasis and increases the secretion of the osteoclastogenic cytokine CXCL12. The resulting phenotype of the Ccl19‐Cre *ERα* ^*fl/fl*^ mice is reduced trabecular bone due to enhanced osteoclastogenesis and decreased bone formation. However, additional studies using more CAR cell–specific conditional knockout mouse models for ERα are required to fully understand the influence of estrogen on CAR cells and effects on bone turnover, both in healthy conditions as well as during skeletal diseases that have a clear female bias, such as rheumatoid arthritis, osteoarthritis, and osteoporosis.

## Disclosures

CO has two patents/patent applications in the field of probiotics and bone health. All other authors state that they have no conflicts of interest.

## Author Contributions

**Julia M Scheffler:** Conceptualization; data curation; formal analysis; investigation; methodology; project administration; validation; visualization; writing – original draft; writing – review and editing. **Karin L Gustafsson:** Data curation; formal analysis; methodology; writing – review and editing. **Aidan Barrett:** Data curation; writing – review and editing. **Carmen Corciulo:** Data curation; methodology; writing – review and editing. **Christina Drevinge:** Data curation; methodology; writing – review and editing. **Alicia M Del Carpio Pons:** Data curation; writing – review and editing. **Piotr Humeniuk:** Data curation; writing – review and editing. **Cecilia Engdahl:** Data curation; writing – review and editing. **Jan‐Åke Gustafsson:** Methodology; resources; writing – review and editing. **Claes Ohlsson:** Methodology; resources; writing – review and editing. **Hans Carlsten:** Funding acquisition; supervision; writing – review and editing. **Marie K Lagerquist:** Methodology; resources; supervision; writing – review and editing. **Ulrika Islander:** Conceptualization; funding acquisition; project administration; supervision; validation; visualization; writing – original draft; writing – review and editing.

## Supporting information


**Appendix S1.** Supplemental Information

## Data Availability

The data that support the findings of this study are available from the corresponding author upon reasonable request.
