# Supplementary material for: ERα Signaling in a Subset of CXCL12‐Abundant Reticular Cells Regulates Trabecular Bone in Mice
Source: JBMR Plus. 2022 Jun 17;6(8):e10657. doi: 10.1002/jbm4.10657 (PMC9382863; doi:10.1002/jbm4.10657)
Supplement: Supplementary file 1 — Appendix S1. Supplemental Information [file JBM4-6-e10657-s001.docx]

Supplemantary table 1: Animals

| **Mouse model** | **Source and catalog #** | **Strain background** | **Providing Laboratory** |
| --- | --- | --- | --- |
| B6.129X1- Gt(ROSA)26Sortm1(EYFP)Cos/J | Jackson  Laboratory, 006148 | C57BL/6J | N/A |
| Ccl19 Cre | N/A | C57BL/6J | Prof. Burkhard Ludewig (Kantonsspital St. Gallen, Switzerland) |
| *ERα*fl/fl | N/A | C57BL/6J | Prof. Jan-Åke Gustafsson (University of Houston, TX, USA) |

Supplementary table 2: Antibodies

| **Epitope/Antigen or product name** | **Source and catalog #** | **Host species** | **Application (IF, FC, MACS)** | **Dilution** |
| --- | --- | --- | --- | --- |
| CD45 MicroBeads, mouse | Miltenyi, 130-052-301 | rat | MACS | 10µl/107cells |
| Ter119 MicroBeads, mouse | Miltenyi,  130-049-901 | rat | MACS | 10µl/107cells |
| Purified Rat Anti-Mouse CD16/CD32 (Mouse BD Fc Block™) | BD Biosciences, 553242 | rat | FC | 1:100 |
| Brilliant Violet 421™ anti- mouse/human CD11b | BioLegend, 101236 | rat | FC | 1:100 |
| Brilliant Violet 421™ anti- mouse CD19 | BioLegend, 115538 | rat | FC | 1:100 |
| Brilliant Violet 510™ anti-  mouse CD8a | BioLegend,  100752 | rat | FC | 1:100 |
| FITC anti-mouse CD11c | BioLegend, 117306 | hamster | FC | 1:100 |
| FITC anti-mouse Ly6A/E (Sca-1) | BioLegend, 108105 | rat | FC | 1:100 |
| PE anti-mouse CD265  (RANK) | BioLegend,  119806 | rat | FC | 1:50 |
| PE anti-mouse CD254 (RANKL) | BioLegend, 510006 | rat | FC | 1:50 |
| PE anti-mouse CD45 | BioLegend, 103106 | rat | FC | 1:100 |
| PE anti-mouse Ter119 | BioLegend, 116208 | rat | FC | 1:100 |
| PE/Cyanine7 anti-mouse  CD3ε | BioLegend,  100320 | hamster | FC | 1:100 |
| PE/Cyanine7 anti-mouse F4/80 | BioLegend, 123114 | rat | FC | 1:100 |
| PE/Cyanine7 anti-mouse Podoplanin | BioLegend, 127412 | hamster | FC | 1:100 |
| PerCP anti-mouse I-A/I-E  (MHCII) | BioLegend,  107624 | rat | FC | 1:100 |
| PerCP anti-mouse Ly-6G/Ly- 6C (Gr-1) | BioLegend, 108426 | rat | FC | 1:100 |
| APC anti-mouse CD115 (CSF-1R) | BioLegend, 135510 | rat | FC | 1:50 |
| APC anti-mouse CD4 | BioLegend, 100412 | rat | FC | 1:100 |
| CD105 (Endoglin) Monoclonal Antibody  (MJ7/18), PE-Cyanine7, eBioscience™ | Thermo fisher scientific,  # 25-1051-82 | rat | FC | 1:100 |

| CD140b (PDGFRB)  Monoclonal Antibody (APB5), APC, eBioscience™ | Thermo fisher scientific,  # 17-1402-82 | rat | FC | 1:100 |
| --- | --- | --- | --- | --- |
| Anti-human EBF3 antibody | Abcam, ab207705 | rabbit | IF | 1:100 |
| Goat anti-Rabbit IgG (H+L) Highly Cross-Adsorbed Secondary Antibody, Alexa  Fluor 647 | Invitrogen, A-21245 | goat | IF | 1:1000 |

Supplementary table 3: Biological Modulators

| **Modulator** | **Source and catalog #** | **Solvent/vehicle** | **concentration** |
| --- | --- | --- | --- |
| Calcein | Sigma, C0875-5G | 2% sodium  bicarbonate solution | 10 mg/ml |
| β-Glycerophosphate disodium salt hydrate | Sigma, G9422-10G | α-MEM | 5mM |
| L-Ascorbic acid 2-phosphate sesquimagnesium salt hydrate | Sigma, A8960-5G | α-MEM | 50µM |
| Insulin | Sigma,  I1507-1mg | dH2O, pH2  with acetic acid | 1mg/ml |
| Dexamethasone | Sigma, D4902-25mg | EtOH | 5mM |
| Indomethacin | Sigma, I7378-10g | DMSO | 200µM |
| M-CSF | R&D systems,  416-ML-010 | PBS | 30ng/ml |
| RANKL | R&D systems, 462-TEC-010 | PBS | 4ng/ml |

# Suppl. Figure 1

A

**bp M**

B

**0.20**


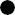

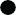

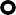


**Uterus weight**

ns

C

**150**

**Gene-expression of *ESR1***

**(normalized to control)**

***Esr1* trab. bone**

ns


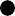

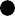

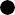

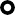

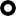


# D

**150**

**Gene-expression of *ESR1***

**(normalized to control)**

***Esr1* deleted 283bp**


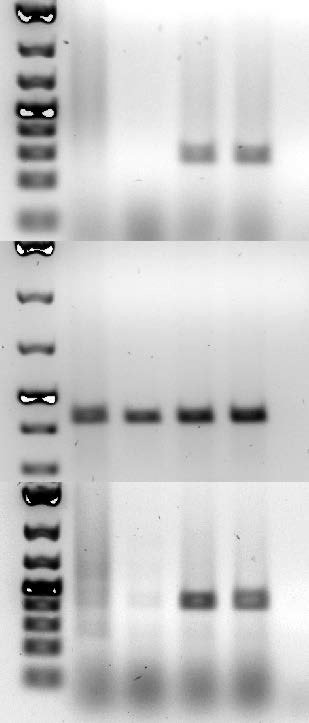
***Esr1* floxed 387bp**

| **1000**  **500** |  |
| --- | --- |
| **1000**  **500** |  |
| **1000**  **500** |  |

**Ccl19-Cre 400bp**

***Esr1* cort. bone**

ns

**0.15**

**0.10**

**[g]**

**0.05**

**0.00**


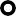


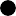


# E

**150**


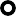


**Gene-expression of *ESR1***

**(normalized to control)**

*ER*αfl/fl

Ccl19-Cre *ER* αfl/fl

***Esr1* BM**

ns

**100**

**50**

**0**


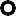


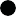


# F

**150**


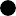

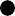

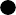

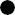

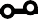

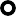

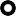


**Gene-expression of *ESR1***

**(normalized to control)**

*ER*αfl/fl

Ccl19-Cre *ER* αfl/fl

***Esr1* OBL**

ns

**100**


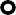


**100**

**100**

**50 50 50**

**0**


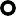
*ER*αfl/fl


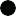
Ccl19-Cre *ER* αfl/fl

**0**


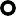
*ER*αfl/fl


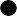
Ccl19-Cre *ER* αfl/fl

**0**


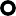
*ER*αfl/fl


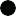
Ccl19-Cre *ER* αfl/fl

G ***Esr1* adipocytes**

**200**


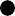


ns

**Gene-expression of *ESR1***

**(normalized to control)**

H

**150**

**Gene-expression of *ESR1***

**(normalized to control)**

***Esr1* BM stroma**

0.0182

**150**


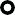

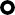

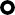


**100**

**100**

**50**

**50**

**0**


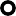
*ER*αfl/fl


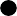
Ccl19-Cre *ER* αfl/fl

**0**


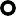
*ER*α

fl/fl

fl/fl


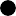
Ccl19-Cre *ER* α

### Supplementary Figure 1:

(A) Genotyping result of two control and two Ccl19-Cre *ERαfl/fl* mice. DNA was extracted from one inguinal lymph nodes and PCR with primers to detects *Esr1* (ERα) floxed, *Esr1* deleted and Ccl19-Cre was run. PCR products are shown on the agarose gel. (B) Uterus weight from both genotypes of 12-week-old mice are shown. (C-H) RT-PCR results for *Esr1* in (C) trabecular bone, (D) cortical bone, (E) bone marrow, (F) *in vitro* cultured osteoblast,

(G) *in vitro* cultured adipocytes and (H) *in vitro* cultured bone marrow stromal cells of control and Ccl19-Cre *ERαfl/fl* mice. n=4-9; Students t-test: p<0.05, ns=non-significant

# Suppl. Figure 2

## BV/TV L5

**30**

0.0248

## Tb.Th L5

**50** 0.0087

## Tb.N L5

ns


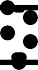

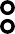


**6**

**20**

**[%]**

**10**

**0**


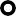
*ER*αfl/fl


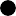
Ccl19-Cre *ER* αfl/fl

**45**

**40**


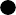


**[µm]**

**35**

**30**

**25**

**0**


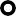
*ER*αfl/fl


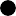
Ccl19-Cre *ER* αfl/fl

**4**

**2**

**[1/mm]**

**0**


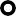
*ER*αfl/fl


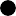
Ccl19-Cre *ER* αfl/fl

# D


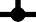

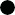

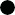


**150**

**100**

**[µm]**

**50**

## Tb.Sp L5

ns

## PINP serum

ns

**80**


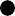

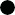

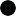

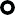


**60**

**[ng/ml]**

**40**

**20**

## CTX-1 serum

**50**


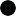

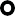


ns

**40**

**30**

**[ng/ml]**

**20**

**10**

**0**


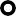
*ER*αfl/fl


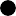
Ccl19-Cre *ER* αfl/fl

**0**


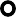
*ER*αfl/fl


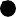
Ccl19-Cre *ER* αfl/fl

**0**


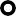
*ER*αfl/fl


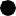
Ccl19-Cre *ER* αfl/fl

# G

**600**

**400**

**[mg/cm^3^]**

**200**

**0**

## Tb. BMD Femur

ns


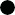

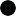

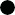

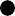


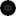
*ER*α+/+


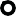
Ccl19-Cre *ER* α+/+

### Supplementary Figure 2:

Lumbar vertebrae L5 of 12-week-old female control and Ccl19- Cre *ERαfl/fl* mice were analyzed by µCT. (A) Trabecular bone volume relative to tissue volume (BV/TV), (B) trabecular thickness, (C) trabecular number and (D) trabecular separation were measured. (E-F) ELISA results of serum from 12-week-old female control and Ccl19-Cre *ERαfl/fl* mice: (E) procollagen type I N propeptide (PINP) and (F) C-terminal type I collagen fragments (CTX-I). (G) pQCT analysis of femurs of female 12- week-old *ERα+/+* and Ccl19-Cre *ERα+/+* mice. Trabecular BMD is shown. n=5-7; (A, C-G) Students t-test, (B) Mann-Whitney; p<0.05, ns=non-significant

# Suppl. Figure 3

## BM cell #

**30**


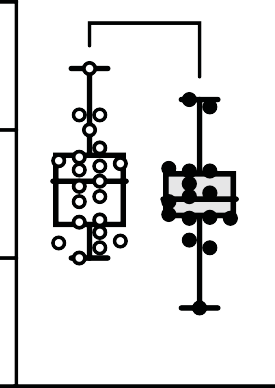


ns

**cells x10^6^ / femur**

**20**

**10**

**0**


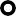
*ER*αfl/fl


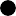
Ccl19-Cre *ER* αfl/fl

## BM granulocytes

ns


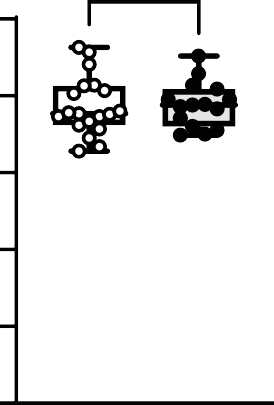
**50**

**% of alive cells / femur**

**40**

**30**

**20**

**10**

**0**


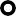
*ER*αfl/fl


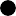
Ccl19-Cre *ER* αfl/fl

## BM macrophages

ns


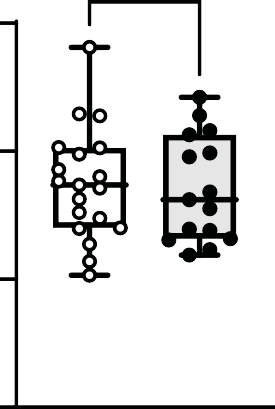
**6**

**% of alive cells / femur**

**4**

**2**

**0**


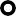
*ER*αfl/fl


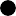
Ccl19-Cre *ER* αfl/fl

## BM monocytes

ns


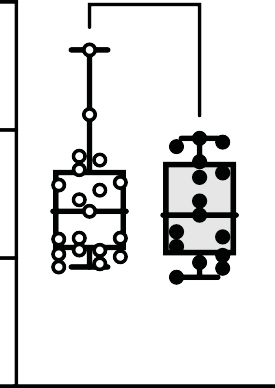
**6**

**% of alive cells / femur**

**4**

**2**

**0**


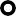
*ER*αfl/fl


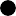
Ccl19-Cre *ER* αfl/fl

E

**0.8**


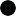

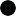

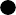

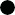

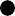

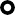

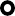

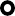

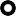

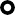


**% of alive cells / femur**

**0.6**

**0.4**

**0.2**

**0.0**


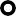


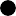


## BM DCs

ns

*ER*αfl/fl

Ccl19-Cre *ER* αfl/fl

### Supplementary Figure 3:

- - 1. Bone morrow cell numbers per femur of the indicated genotype. n=16-21; (B-E) Flow cytometry was performed on bone marrow cells of 12-week-old female control and Ccl19-Cre *ERαfl/fl* mice. Results are shown as percentage of alive cells from one femur: (B) granulocytes, (C) macrophages, (D) monocytes, (E) Dendritic cells (DCs). n=8-19; (A, C, E) Students t - test, (B, D) Mann-Whitney test; p<0.05 ns= not significant

# Suppl. Figure 4

A

**2.0**


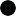

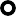


**MAR**

ns

B ***Tnfrfs11b* cort. bone**

**150**


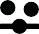

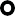


0.0120

**Gene-expression of *TNFRSF11***

**(normalized to control)**

C

**150**

**Gene-expression of *TNFSF11***

**(normalized to control)**

***Tnfsf11* cort. bone**

0.0488

**1.5**

**[µm/d]**

**1.0**

**0.5**

**100**

**50**

**100**

**50**


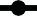

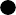

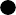

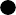

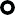


**0.0**


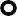


*ER*αfl/fl

Ccl19-Cre *ER* αfl/fl

**0**

*ER*αfl/fl

Ccl19-Cre *ER* αfl/fl

**0**

*ER*αfl/fl

Ccl19-Cre *ER* αfl/fl

D ***Tnfrsf11b*/*Tnfsf11***

## cort. bone

**3**

ns

E

**200**

**Gene-expression of *Bglap***

**(normalized to control)**

***Bglap* cort. bone**

ns

F

**150**

**Gene-expression of *Runx2***

**(normalized to control)**

***Runx2* cort. bone**

**150**

**ratio fold changes**

**2**

**100**

**1**

**50**

**100**

**50**

ns

**0**

*ER*αfl/fl

Ccl19-Cre *ER* αfl/fl

**0**

*ER*αfl/fl

Ccl19-Cre *ER* αfl/fl

**0**

*ER*αfl/fl

Ccl19-Cre *ER* αfl/fl

### Supplementary Figure 4:

(A) Mineral apposition rate (MAR) in the distal endocortical region of femurs of 12 week-old female wildtype and Ccl19-Cre *ERαfl/fl* mice was analyzed by calcein incorporation. (B-F) Gene expression in cortical bone (tibia bone shaft without bone marrow) of 12-week old female control and Ccl19-Cre *ERαfl/fl* mice was analyzed. Results are shown in percentage normalized to wildtype mean expression: (B) *Tnfrsf11b* (OPG), (C) *Tnfsf11* (RANKL), (D) ratio of *Tnfrsf11b* and *Tnfsf11* was calculated, (E) *Bglap* (osteocalcin) and (F) *Runx2*. n=4-6; (A-D,F) Students t-test, (E) Mann-Whitney test: <0.05, ns= not significant

# Suppl. Figure 5

## BM B cells

1. **B cells RANKL MFI**
2. **RANKL pos. B cells**

**25** ns **60** ns

**% of alive cells / femur**

**20**

**[geometric mean]**

**40**

**15**

**10**

**20**

**5**

**0.8** ns

**0.6**

**% of alive cells / femur**

**0.4**

**0.2**

**0**

*ER*αfl/fl

Ccl19-Cre *ER* αfl/fl

**0**

*ER*αfl/fl

Ccl19-Cre *ER* αfl/fl

**0.0**

*ER*αfl/fl

Ccl19-Cre *ER* αfl/fl

## BM T cells

**5**

ns

**% of alive cells / femur**

**4**

**3**

**2**

**1**

**0**

*ER*αfl/fl

Ccl19-Cre *ER* αfl/fl

E

**150**

**[geometric mean]**

**100**

**50**

**0**

## T cells RANKL MFI

ns

*ER*αfl/fl

Ccl19-Cre *ER* αfl/fl

F

**1.0**

**% of alive cells / femur**

**0.8**

**0.6**

**0.4**

**0.2**

**0.0**

## RANKL pos. T cells

ns

*ER*αfl/fl

Ccl19-Cre *ER* αfl/fl

### Supplementary Figure 5:

Flow cytometry was performed on bone marrow cells of 12-week-old female control and Ccl19-Cre *ERαfl/fl* mice. Results are shown as percentage of alive cells from one femur: (A) bone marrow B cells, (B) RANKL expression on B cells as mean fluorescent intensity (MFI),

(C) percentage of RANKL positive B cells, (D) bone marrow T cells, (E) RANKL expression on T cells as mean fluorescent intensity (MFI) and (F) percentage of RANKL positive T cells. n=7-9; Students t-test: p<0.05 ns= not significant

# Suppl. Figure 6

## LN of EYFP Ccl19-Cre *ERα*^+/+^

**alive single cells**

## BM of EYFP Ccl19-Cre *ERα*^+/+^

**alive single cells**

### Supplementary Figure 6:

- - 1. Gating strategy for EYFP positive fibroblastic reticular cells (FRC) in inguinal Lymph node (LN) of an EYFP Ccl19-Cre *ERα+/+* control mouse. Stromal cells were enriched via negative MACS selection for CD45 and Ter119. (B) Gating strategy for EYFP positive CAR cells in bone marrow of an EYFP Ccl19-Cre *ERα+/+* control mouse.
